# Chromatin differentiation of white blood cells decreases DSB damage induction, prevents functional assembly of repair foci, but has no influence on protrusion of heterochromatic DSBs into the low-dense chromatin

**DOI:** 10.1093/jrr/rrt194

**Published:** 2014-03

**Authors:** Martin Falk, Emilie Lukasova, Iva Falkova, Lenka Stefancikova, Lucie Jezkova, Alena Bacikova, Marie Davídková, Alla Boreyko, Evgeny A. Krasavin, Stanislav Kozubek

**Affiliations:** 1 Institute of Biophysics, Academy of Sciences CR, Brno, Czech Republic; 2 Joint Institute for Nuclear Research, Dubna, Russia; 3 Institute of Chemical Technology Prague, Prague, Czech Republic; 4 Nuclear Physics Institute, Academy of Sciences of CR, Rˇež, Czech Republic

**Keywords:** DNA double-strand breaks (DSB), DSB repair, white blood cells differentiation, higher-order chromatin structure, ionizing radiations of different quality, ionizing radiation-induced repair foci (IRIF)

## Abstract

**Purpose:** Higher order chromatin structure progressively changes with cell differentiation and seems to play an important role in DNA double-strand break (DSB) induction and repair (reviewed in [1]). We compared DNA damage in heterochromatin (Hc) upon the action of qualitatively different radiations. We also studied, how is the sensitivity to DSB induction, assembly of repair foci and processing of DSBs influenced by the differentiation-induced changes in chromatin structure and composition.

**Materials and methods:** Formation, localization (relative to higher-order chromatin domains) and mutual colocalization of γH2AX and p53BP1 repair foci have been studied together with DSB repair kinetics in spatially fixed human skin fibroblast and differently differentiated white blood cells (WBC) irradiated with gamma rays, protons of different energies [2, 3], and ^20^Ne ions (submitted). Immunostaining and ImmunoFISH were used in combination with high-resolution confocal microscopy [2, 3] and living cell imaging [4].

**Results:** We found that less DSBs appear in Hc after irradiating cells with gamma rays and protons but not ^20^Ne ions (preliminary results). In addition, contrary to γ-irradiated human skin fibroblasts and lymphocytes, mature granulocytes neither express DSB repair proteins nor form functional repair foci [5]. At least some DSB repair proteins (e.g. 53BP1) are expressed and γH2AX foci still occur in immature granulocytes and monocytes [2, 5]; however, the colocalization of γH2AX with 53BP1 is low and the majority of DSBs are not repaired. Despite this fact, γH2AX foci protrude from Hc into nuclear subcompartments with low chromatin density. Our living cell observations suggest that 53BP1 can penetrate into the interior of dense Hc domains only after their decondensation [2].

**Conclusions:** We show that Hc is less sensitive to DSB induction by gamma rays but not heavy ions; lower Hc hydratation and higher protein density (when compared with euchromatin) probably reduce formation of free radicals and increase their sequestration, respectively. This mechanism can protect cells against the indirect effect of ionizing radiation (marked for gamma rays and protons but not heavy ions). Hc features, however, preclude DSB repair, which is best illustrated by its absence in differentiated WBC but not their immature precursors. The protrusion of Hc-DSBs into low-density chromatin nuclear subdomains, however, appears also in differentiated WBC, so the process might simply follow physical forces (e.g. as suggested by M Durante's group).

There is no Clinical Trial Registration number.
